# Scientist Spotlights: Online assignments to promote inclusion in Ecology and Evolution

**DOI:** 10.1002/ece3.6849

**Published:** 2020-10-08

**Authors:** Samantha Brandt, Sehoya Cotner, Zoe Koth, Suzanne McGaugh

**Affiliations:** ^1^ Department of Biology Teaching and Learning University of Minnesota Twin Cities Minneapolis MN USA; ^2^ Department of Ecology Evolution and Behavior University of Minnesota Twin Cities Saint Paul MN USA

**Keywords:** ecology, evolution, inclusive teaching, role models, Scientist Spotlights

## Abstract

Scientific disciplines face large diversity challenges, with the fields of ecology and evolution being among the most homogeneous—specifically with respect to race and ethnicity. These problems have been recently compounded by large‐scale racial unrest, highlighting some of the underlying disparities that have led to these diversity challenges, and a global pandemic, which, by moving instruction online, has created new challenges for inclusive teaching. Among the inclusive‐teaching techniques that can be implemented during remote instruction are Scientist Spotlights—role‐model interventions that use available online materials to highlight the work of scientists representing multiple axes of diversity. We report here on the implementation of Scientist Spotlights in two courses, both of which emphasize ecology and evolution. We conclude with sample resources and suggestions for adopters.

## Graphical abstract

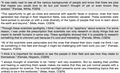

## 1 INTRODUCTION

In order to address the current need to train more science, technology, engineering, and math (STEM) professionals, we must address the diversity crisis in STEM disciplines. Specifically, these disciplines are plagued by lower performance, participation, and retention of students characteristically underrepresented in STEM—women, certain ethnic and racial minorities, and first‐generation college students (Chen, 2013; Graham et al., 2013; Hill et al., 2010; President's Council of Advisors on Science & Technology, 2012; National Academies of Sciences, Engineering, and Medicine, 2018). The reasons for these disparities are complicated, and both context‐ and identity‐dependent, however, suggested causes include discrepancies in preparation (Kugler et al., 2017), depersonalized and didactic teaching methods (Malone et al., 2009; Mervis, 2010; National Academies of Sciences, Engineering, and Medicine, 2018), stereotype threat (Cohen, 2006; Steele, 1997; Walton & Cohen, 2007), implicit biases (Moss‐Racusin et al., 2012; Staats, 2015), and a lack of role models to whom aspiring scientists can relate (Herrmann et al., 2016; O'Brien et al., 2017; Stout et al., 2011). Collectively, these reasons constitute the “opportunity gap” for full representation in STEM. As educators and practicing scientists, we can counter this opportunity gap by: bridge programs and supplemental instruction (i.e., to mitigate discrepancies in preparation; (Ballen & Mason, 2017; Cooper et al., 2017)); inclusive, empathetic teaching practices (Dewsbury & Brame, 2019; Tanner, 2013); implicit‐bias training (Chang et al., 2019; Jackson et al., 2014); and role‐model interventions (Ramsey et al., 2013; Van Camp et al., 2019).

The work described herein focuses on role models in STEM—specifically in ecology and evolution (E&E), and specifically in a remote‐teaching environment. E&E are especially homogeneous fields, historically, and currently being the domain of White men. For example, a study of authorship in the journal *Ecology* showed that, of 922 authors in 2011, 72% were men; further, 33% of first authors, and 21% of last authors, were women (Martin, 2012). Fisheries science is especially homogeneous, with men representing over 70% of tenure‐track faculty; 88% of these faculty are White (Arismendi & Penaluna, 2016). Similar trends have been documented in evolutionary biology (Graves, 2019; Schroeder et al., 2013). Other studies have shown that students (including those in higher education) picture scientists as older White men in laboratory coats (Barman, 1999; Long et al., 2001; Schinske et al., 2015; Steinke et al., 2007); if a student's identity or identities differ from this mental image, they may have difficulty seeing their possible selves in science (Chang et al., 2011; Schmader et al., 2004).

Underrepresented students failing to identify with ecologists and evolutionary biologists is a known problem, so we can explore the potential of role‐model interventions in our classrooms (Shin et al., 2016; Van Camp et al., 2019). Role‐model interventions can take many forms, from the simple (Schinske et al., 2016; Yonas et al., 2020) to the more complex (Barnes et al., 2017). For example, in order to mitigate student perceptions of the conflict between science and religion, Jim Elser of Arizona State University invited a scientist—who also identified as religious—to speak, via videoconference, to his introductory‐biology class (Barnes et al., 2017); the number of students who perceived a conflict between science and religion decreased after this experience. These findings suggest that students who do not identify with evolutionary biology on religious grounds may benefit from these interventions. Both Schinske et al. (2016) and Yonas et al. (2020) describe small, online “Scientist Spotlight” assignments designed to introduce students to a diverse group of scientists working in the domain of course topics. Specifically, students learn about the work and life experiences of a diverse array of scientists, many with counter‐stereotypical (e.g., LGBTQ, non‐White) expressed identities. Typically, the science content takes a secondary role to the scientist's personal story. At the end of the courses described, students participating in Spotlights express an ability to relate more to scientists (Schinske et al., 2016; Yonas et al., 2020), and use more counter‐stereotypical examples in their descriptions of who does science (Schinske et al., 2016). Yonas et al. (2020) add to the dialogue by urging the incorporation of “hidden identities” into our concept of diversity—for example, by including scientists that are religious, politically conservative, etc.

In sum, past research has shown that it is possible to change students' perceptions of who can do science. The current work adds to prior work by focusing on students and role models in E&E (specifically, General Zoology and Evolution & Biology of Sex) courses and asks the following questions:

- Can these Scientist Spotlight assignments mitigate some of the diversity concerns that characterize E&E?
- Do students specify being able to relate to scientists based on gender, race/ethnicity, or other categorical descriptors of identity?

We use survey data to respond to these questions, and, given our numerical limitations, we emphasize qualitative data in our assessment of the Scientist Spotlights.

Our specific aim with the current paper is primarily to present to our colleagues in E&E a simple, adaptable course addition that can complement any of our course topics while introducing students to a diverse group of possible role models. We also conclude by providing potential adopters with resources for incorporating Scientist Spotlights into their own courses. We conclude with suggestions and resources for adopters.

## 2 SCIENTIST SPOTLIGHTS FOR ECOLOGY AND EVOLUTION

### 2.1 Student population

We describe Scientist Spotlight interventions in two Biology courses at the University of Minnesota. *The Evolution and Biology of Sex* course (Biology 1003) is a nonmajor introductory‐biology course emphasizing evolution from the lens of sex—reproduction, sexual orientation, mating systems, etc. Two‐hundred and thirty‐four students took part in the assignments, and assessments described herein. This was the second iteration of the Scientist Spotlight intervention in Biology 1003 (see Yonas et al. (2020) for additional details from the initial implementation). *General Zoology* (Biology 2012) is an organismal biology course, emphasizing ecology and evolution throughout, for students majoring in multiple natural‐sciences fields, from Ecology, Evolution and Behavior to Fisheries and Wildlife. Ninety‐one students took part in the assignments, and assessments we describe.

### 2.2 Scientist Spotlight assignments

In both courses, Scientist Spotlights were posted online throughout the course (Appendix S1 includes links to several example spotlights), and students were instructed to complete each assignment by its given deadline. Eight Spotlight assignments were used throughout the semester. This number was chosen so that there was enough exposure to the content for it to have an effect on students and for the assignments to become a reliable and anticipated part of the course, but not so many so as to make the workload overwhelming. In each case, students viewed a video or listened to a podcast of a current scientist discussing either their work, their experiences as a scientist, or both. Afterward, students were asked to reflect on what they learned from the Spotlight and how it influenced their perceptions of real‐world scientists. These assignments were graded on completion alone.

Scientists were chosen based on their field of study (and how it relates to the course topics), as well as their identity (or identities) along multiple axes of diversity—race/ethnicity, gender, ability, sexual orientation, religiosity, etc., and their status as currently active scientists. For example, they can learn about the work of Dr. Delbert André Green, an associate professor of Ecology and Evolutionary Biology at the University of Michigan. Dr. Green studies the genetics and evolution of migration, with monarch butterflies as a model system. Some of the scientists address their underrepresented identities head‐on. For example, Dr. Mimi Koehl of the University of California Berkeley tells a story about being dyslexic and how that has affected her work in science. And Dr. Jessica Ware, of Rutgers University‐Newark, discusses both her research on cockroaches and her experiences being an LGBTQ mother to LGBTQ children.

As part of each Spotlight, students answered open‐ended questions about the featured scientist and their work. A consistent open‐ended question, included in every Spotlight, was “What do these materials tell you about the types of people who do science?” In each course, these Spotlight assignments constituted a small amount of points (<5%) toward a student's overall grade.

### 2.3 Assessment of the Scientist Spotlights

Students were invited, via email, to complete short pre‐ and postcourse surveys, administered online. Both pre‐ and postcourse surveys met the research aims of multiple investigators and included items not necessarily relevant to this study. For this study, students were asked to indicate the number of scientists they knew of that they found to be relatable. *Relatability* is defined here as the ability for a person to identify with figures such as those featured in the Scientist Spotlights. This question could be answered from predefined ranges of 0, 1, 2–5, 6–10, and 10+. The postcourse survey repeated this question. Postcourse surveys also asked the students to reflect on the specific Scientist Spotlights. Students were first asked if they had viewed the materials and then to identify which ones had been the most engaging (“Recall the Scientist Spotlight materials from this semester. I've included names and some keyword prompts below. Please comment on the level to which you were engaged by the materials,” followed by a list of the scientists) and relatable (“For whatever reason or reasons, which of the following Scientist Spotlight materials did you connect with, or relate to, the most?” followed by a list of the Spotlighted scientists). Students were also provided with free‐response space to explain their answers (“Please explain your answer to the question above.”).

Demographic data were either self‐reported (e.g., gender, sexual orientation) or institutional (e.g., race or ethnicity, generation in college). After surveys were completed, the data were de‐identified for analysis. Students that only completed one of the surveys were excluded from data analysis. The University of Minnesota Institutional Review Board (IRB) found this research to pose minimal risk, and thus, it is considered exempt from IRB review; however, all student data discussed here were shared with each student's informed consent.

Because a mixed‐methods approach was used to collect both qualitative and quantitative data, different methods were used to assess the impact of the Scientist Spotlight assignments in these two E&E courses. Specifically, we used concurrent triangulation (sensu Warfa, 2016) as follows:

- *Quantitative analysis*. The constrained‐choice survey items allowed students to select the number of scientists with whom they could relate. By matching students' pre‐ and postcourse responses, we can report on changes in the number of scientists to whom they can relate before and after engaging with the Spotlights. Due to the small size of our final, matched dataset, we avoid any statistical comparisons beyond descriptive data.
- *Qualitative analysis*. While more extensive coding has been used in previous work (Yonas et al., 2020), for this assessment we focus on codes specifically relating to student perceptions of the Spotlight scientists' relatability, and how student perceptions of scientists were swayed by these interventions. Specifically, all student comments from the postcourse surveys were analyzed independently by two researchers (ZK and SB); the first objective was to identify student comments that specifically addressed how the student related to scientists and develop codes incorporating similar sentiments (specifically using a modified “cutting and sorting” technique for qualitative coding). After the first round of coding, the two researchers met to discuss any areas of disagreement and reach consensus. Only consensus codes, pertaining to scientist relatability, are shared below. After coding was completed, students' comments were aligned with student demographics—self‐reported and institutional—to arrive at the student descriptors presented in our discussion below.

## 3 DO SCIENTIST SPOTLIGHTS INCREASE RELATABILITY?

### 3.1 Quantitative findings

**FIGURE 1:**
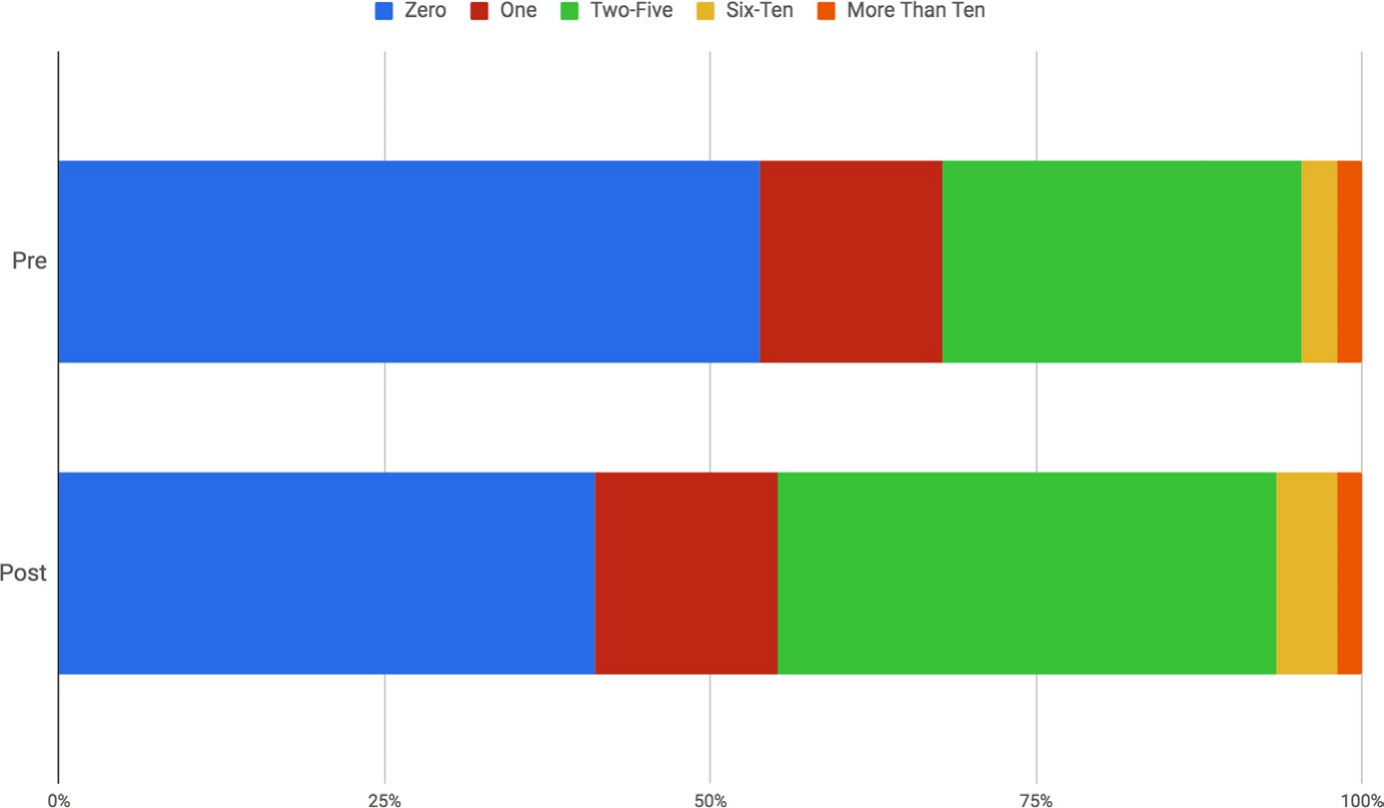
The number of scientists students relate to before and after Scientist Spotlights in Biology 1003 (*n* = 214)

**FIGURE 2:**
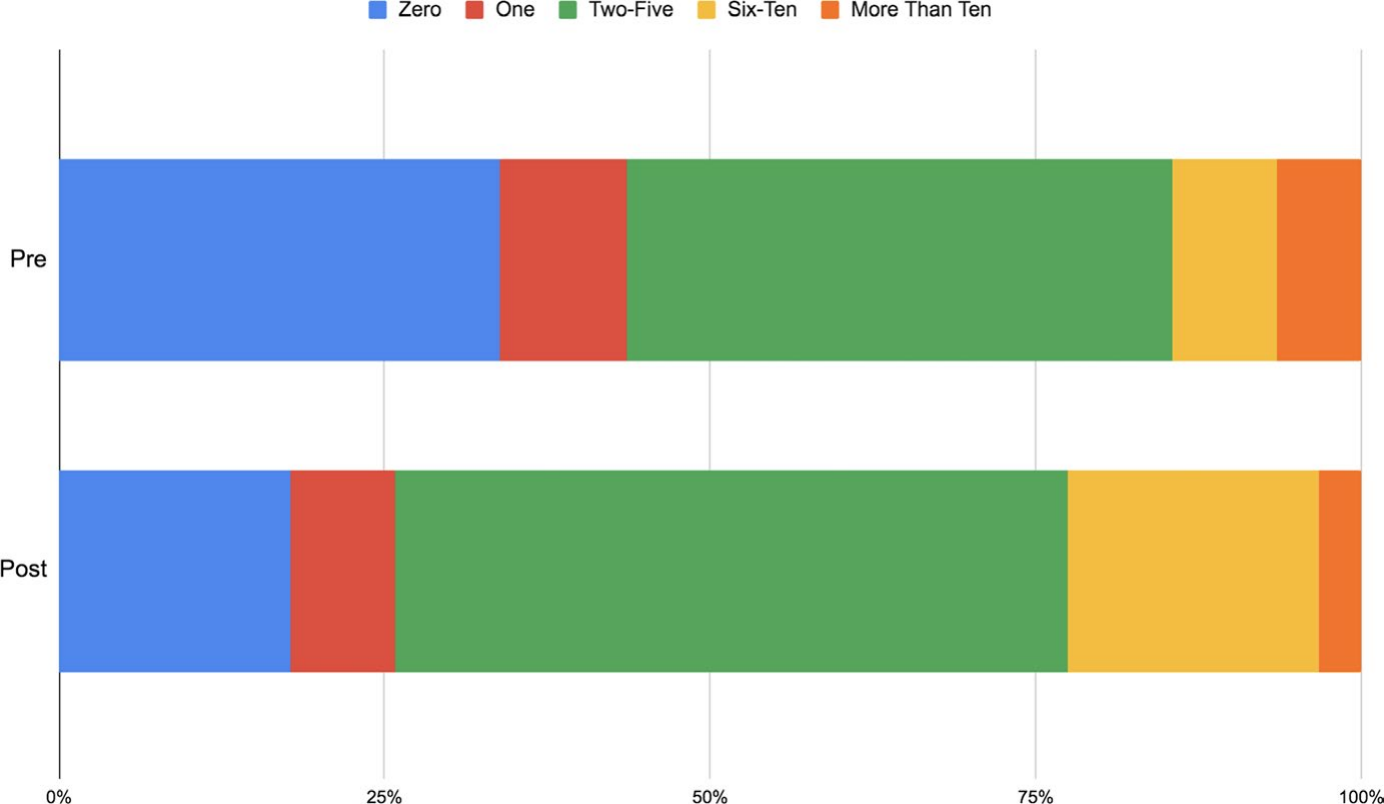
The number of scientists students relate to before and after Scientist Spotlights in Biology 2012 (*n* = 62)

**FIGURE 3:**
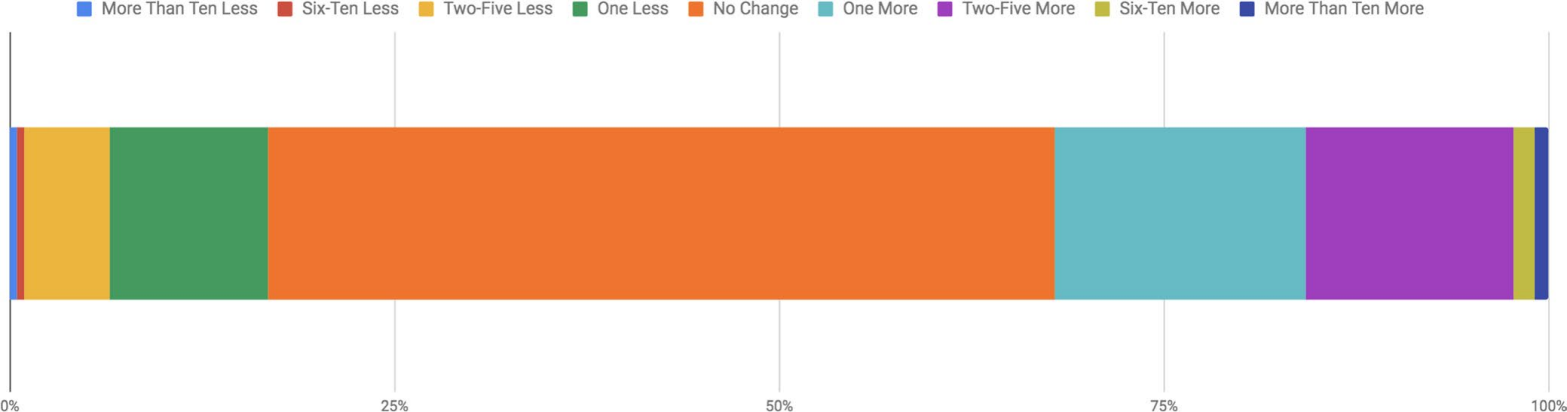
The change in the number of scientists students relate to following the Scientist Spotlights in Biology 1003 (*n* = 214)

**FIGURE 4:**
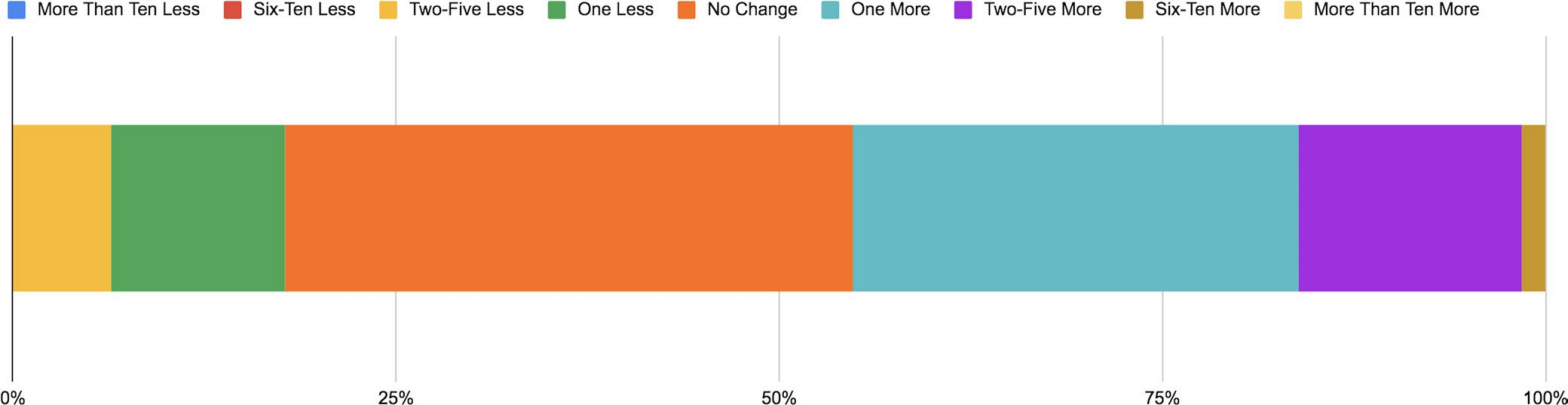
The change in the number of scientists students relate to following the Scientist Spotlights in Biology 2012 (*n* = 62)

Change in students' ability to relate to scientists was measured using the number of scientist students reported they knew and could relate to across the pre‐ and postcourse surveys (Figures 1, 2, 3, 4). On the precourse survey in Biology 1003, 50.8% of students reported not knowing or relating to any scientists (Figure 1), with female students reporting a slightly higher inability to relate to scientists (+6.7%) than male students. Following the Scientist Spotlight assignments, the students' overall inability to relate to scientists was reduced to 40.75%, with female students showing a higher ability to relate to scientists, compared to their male counterparts (+2.9%). Overall, both gender groups reported higher rates of relatability in postclass responses. Due to the nature of this assessment, we are unable to identify the extent to which the Spotlights themselves increase relatability to scientists, if at all.

### 3.2 Qualitative findings

The majority of students in both courses responded to the open‐ended prompts about engagement and relatability. We focus here on the answers to the relatability prompt.

Many students responded positively to the podcasts *in general*, with statements such as “To be honest I loved most of them, I actually looked forward to completing this assignment because they were all so good and I learned a lot” (Female, White, Continuing‐Generation College Student (CGEN)). Some voiced an appreciation for the exposure to diversity: “I just liked the podcasts in general. I like the purposeful exposure to different types of people and lifestyles while still keeping science as the common denominator” (Female, White, CGEN). Others noted specific aspects of a featured scientist's identity: “I thought Scott Edwards was engaging because he was studying bird evolution, and was a POC scientist in the natural resources field, which is rarer (Female, Asian, First‐Generation College Student (FGEN)).” And still others aligned their appreciation of featured scientists' identities with the student's identities: “I related most to the scientists with backgrounds similar to mine, especially women” (Female, White, First‐generation college student); and “I feel like I relate to indigenous women scientists” (Female, Native American, FGEN).

**FIGURE 5:**
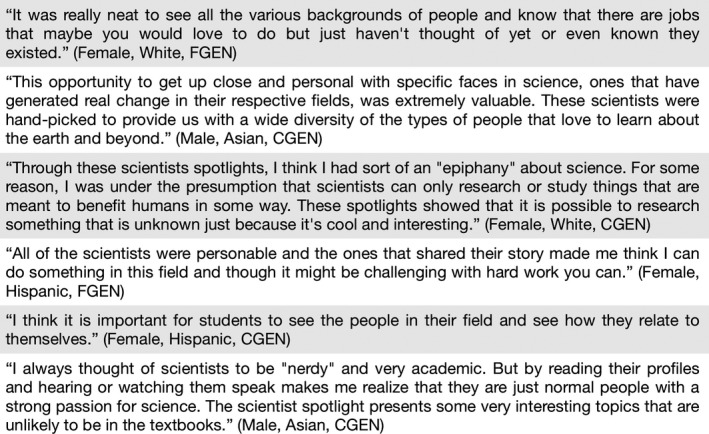
Sample student comments, in response to the open‐ended survey question asking for elaboration on their relatability choices (i.e., which scientists the students found to be most relatable). Available (albeit limited) specifics about each respondent are given in parentheses. FGEN = first‐generation college student (neither parent attended college): CGEN = continuing‐generation college student (one or more parent attended college)

Additional student comments, aligned with certain student identities (gender, race/ethnicity, generation in college), are presented in Figure 5.

## 4 CONCLUSION AND RECOMMENDATIONS

In implementing these Scientist Spotlights, our hope was that exposure to the stories of diverse scientists would allow students to form counter‐stereotypical views of who does science. Progress in this area is likely to differentially impact students from groups traditionally underrepresented in STEM, and for our purposes, in ecology and evolution. Specifically, students who may have previously viewed themselves as outside the stereotypical scientist identity might better relate to the field and more easily see themselves as scientists in the present or future. At a minimum, we hoped to increase the number of students who found scientists (and therefore science itself) relatable. Our specific questions were as follows:

- Can these Scientist Spotlight assignments mitigate some of the diversity concerns that characterize E&E?
- Do students specify being able to relate to scientists based on gender, race/ethnicity, or other categorical descriptors of identity?

We can answer, in the affirmative, both questions. We are encouraged by the fact that, over the course of the semester, student ability to relate to scientists increased measurably, if not dramatically. Due to the nature of our implementation, we cannot control for other factors that may have contributed to this gain, nor can we pinpoint aspects of the Spotlights that may have been especially meaningful. However, student comments do shed light on these relatability changes; specifically, several students—including those from groups underrepresented in STEM—expressed an appreciation for the inclusion of diverse scientist identities, and several aligned themselves with specific scientists based on the scientists' expressed identities. Thus, we feel that these assignments can be a valuable component of an instructor's attempts at making their Ecology or Evolution course more inclusive. Further, they can be done completely online, lending themselves well to the current demand for remote instruction that fosters diversity, equity, and inclusion.

Because these assignments are compelling, easy to implement, and not intrusive (i.e., they don't need to detract from primary content‐related goals of a course), they are a simple foray into inclusive teaching. Perhaps equally as important, providing these assignments is an easy way to signal an interest in diversity and inclusion, possibly opening the door to continued dialogue between the instructor and students who may typically feel less comfortable—for whatever reasons—approaching a science faculty member. While we by no means see these Spotlights as being capable of solving the capacious diversity challenges in ecology and evolution, we do consider them a valuable component of inclusive teaching and a step in the right direction.

For our colleagues interested in implementing these, or similar, assignments in their courses, we offer the following suggestions:

- Incorporate these assignments throughout the course, ideally beginning them during the first 1–2 weeks of the term. This way the Spotlights will be viewed as integral to the course, rather than an add‐on and not truly representative of an interest in inclusive teaching.
- Use existing materials! We've provided (Appendix S1) some suggestions, and options abound:
  - Scientist Spotlights (scientistspotlights.org) is a searchable resource for “integrating themes of diversity and inclusion while teaching course content.”
  - Story Collider (storycollider.org) is a searchable resource for “true, personal stories about science.”
  - Project Biodiversify (https://projectbiodiversify.org/) curates “tools for promoting diversity and inclusivity in biology classrooms.”
  - PBS' NOVA: Secret Life of Scientists & Engineers (https://www.pbslearningmedia.org/collection/nvslos/) “profiles today's leading scientists—and shows what they're like when the lab coats come off—showing viewers a human side of science that many students can relate to.” There is also a special subsection entitled Diversity in STEM (https://www.pbslearningmedia.org/collection/secret‐life‐of‐scientists‐and‐engineers‐diversity‐in‐stem/)
- Finally, solicit student feedback throughout. Student populations vary, and depending on the nature of the course, the discipline, and the institutional profile, different groups of students may feel marginalized. For example, in our evolution‐focused nonmajor course, we felt that helping students reconcile the perceived conflict between science and religion was important. In other courses, instructors will be aware of (or will learn from their students) which identities are most in need of representation through these Spotlights.

## CONFLICT OF INTEREST

The authors declare no competing interests.

## AUTHOR CONTRIBUTIONS

**Samantha Brandt:** Data curation (lead); formal analysis (equal); visualization (equal); writing – original draft (equal); writing – review & editing (equal). **Sehoya Cotner:** Conceptualization (lead); project administration (lead); resources (equal); supervision (equal); writing – original draft (equal); writing – review & editing (equal). **Zoe Koth:** Data curation (equal); formal analysis (equal); writing – original draft (equal); writing – review & editing (equal). **Suzanne McGaugh:** Conceptualization (equal); project administration (equal); writing – original draft (equal); writing – review & editing (equal).

## Supporting information

Appendix S1

## Data Availability

Due to our use of human subjects in this research, we are only able to provide de‐identified, aggregate data upon request.
