# Supplementary material for: Scientist Spotlights: Online assignments to promote inclusion in Ecology and Evolution
Source: Ecol Evol. 2020 Oct 8;10(22):12450–6. doi: 10.1002/ece3.6849 (PMC7679542; doi:10.1002/ece3.6849)
Supplement: Supplementary file 1 — Appendix S1 [file ECE3-10-12450-s001.pdf]

Name	Position	Area of Study	Resources
**Dr. Maydianne Andrade**	Professor and Vice-Dean at the University of Toronto - Scarborough	Reproductive behaviors and developmental plasticity in animals, especially arachnids	https://vimeo.com/16475149
**Dr. Shane Campbell-Staton**	Assistant Professor of Biology at University of California - Los Angeles	Integrates physiology, genomics, and field work to study how complex phenotypes respond to anthropogenic climate change	https://www.youtube.com/watch?v=bqColbiPlWs<br>https://www.youtube.com/watch?v=J-_mzMOUN5A
**Dr. Prosanta Chakrabarty**	Curator of Ichthyology and Associate Professor at Louisiana State University	Bioluminescent systems and historical biogeography of freshwater fishes	https://www.ted.com/talks/prosanta_chakrabarty_clues_to_prehistoric_times_found_in_blind_cavefish?language=en#t-31598<br>https://www.ted.com/talks/prosanta_chakrabarty_four_billion_years_of_evolution_in_six_minutes?language=en<br><br>https://www.youtube.com/watch?v=IL-9bplZa0E<br>https://www.youtube.com/watch?v=wSJPSnWvaVY
**Dr. Eric Edsinger**	Research Scientist at the Salk Institute for Biological Studies	Cephalopod neurobiology	https://www.sacnas.org/team-details/eric-edsinger/
**Dr. Scott Edwards**	Professor of Organismic and Evolutionary Biology and Curator of Ornithology in the Museum of Comparative Zoology at Harvard University	Efforts to unite genomics and natural history of birds, including phylogenetics and genome evolution	https://www.youtube.com/watchtime_continue=192&v=3R7vIjY2pwk
**Dr. Lauren Esposito**	Assistant Curator and Schlinger Chair of Arachnology at the California Academy of Sciences, and the co-founder/director of a science, education, and conservation non-profit called Islands & Seas. She is also the founder of 500 Queer Scientists.	Evolution of scorpions and their venom	https://www.calacademy.org/learn-explore/science-heroes/lauren-esposito<br>https://untamedscience.com/entomologists/lauren-esposito-entomologist/<br>https://www.youtube.com/watch?v=2ifeU-Zxi5o
**Dr. Kory Evans**	Assistant Professor in Department of Biosciences at Rice University	Evolution of phenotypic diversity and innovation by studying cranial facial structures in neotropical fish	https://www.youtube.com/watch?v=UHtyoTOAyg8
**Dr. Temple Grandin**	Professor of Animal Science at Colorado State University	Livestock welfare and safety	https://www.youtube.com/watch?v=9Ty6vj3aPxM<br>https://www.youtube.com/watch?v=C8xaW84b7U8<br>https://www.youtube.com/watch?v=6wxewRynV3g
**Dr. Delbert André Green**	Assistant Professor in Ecology and Evolutionary Biology at University of Michigan	Mechanisms that constrain or promote biodiversity and utilizes monarch butterflies as a model to understand the genetic basis and evolution of migration	https://lsa.umich.edu/eeb/news-events/all-news/search-news/on-u-m-gateway--monarch-butterflies-rely-on-temperature-sensitiv.html<br>https://lsa.umich.edu/lsa/news-events/all-news/search-news/rewriting-the-monarch-story.html
**Dr. Mimi Koehl**	Professor of Integrative Biology at University of California - Berkeley	Marine biology, physics, fluid and solid mechanics, and ecology	https://www.youtube.com/watch?v=4mdz88JvYEg
**Dr. Mohamed Noor**	Professor of Biology and Dean of Natural Sciences at Duke University	Evolution and genetics, particularly on how species split and stay separated to form more biodiversity	https://www.youtube.com/watch?v=TtT6g043WdQ<br>https://today.duke.edu/2009/02/noor.html

Name	Position	Area of Study	Resources
**Dr. C. Brandon Ogbunu**	Assistant Professor of Biology at Brown University	Evolutionary systems, experimental evolution, and mathematical modeling to understand the evolution of drug resistance due to complex genetic and environmental interactions	https://www.youtube.com/watch?v=crGgChrrKXE https://www.evolutionsociety.org/content/new-faculty-profiles/new-faculty-profile-c-brandon-ogbunu.html https://www.wired.com/story/james-watson-and-scientific-racism/
**Dr. Sheila Patek**	Associate Professorof Biology at Duke University	Interface of evolution and physics by researching astonishingly fast strikes of mantis shrimp and other examples of extremely quick or powerful movements in the natural world	https://www.youtube.com/watch?v=QRYFI8GrDHs https://www.pbs.org/newshour/show/why-knowledge-for-the-pure-sake-of-knowing-is-good-enough-to-justify-scientific-research
**Dr. Michael Purugganan**	Professor of Biology at New York University	Evolution of domestication process, especially in rice and date palms. He also studies the ecological and evolutionary genomics of plant environmental adaptation and the evolution of social behavior in a model protist.	https://www.youtube.com/watch?v=uyYnMk3auCI https://www.rappler.com/thought-leaders/academe/41920-purugganan-why-i-support-golden-rice
**Dr. Lauren Sallan**	Assistant Professor of Earth and Environmental Science and Biology at University of Pennsylvania	Species persistence, diversification, and extinction on a palenotological and macroevolutionary scale.	https://www.ted.com/talks/lauren_sallan_a_brief_tour_of_the_last_4_billion_years_dinosaurs_not_included https://www.ted.com/talks/lauren_sallan_how_to_win_at_evolution_and_survive_a_mass_extinction
**Dr. Sonia Tikoo**	Assistant Professor of Geophysics at Stanford University	Lunar paleomagnetism and studies the physical effects of impact events on planetary surfaces	https://www.pbs.org/video/day-the-dinosaurs-died-rooax3/ https://www.netflix.com/title/81121175 https://www.dailymotion.com/video/x6d8tba
**Dr. Jenny Tung**	Associate Professor in the Departments of Evolutionary Anthropology and Biology at Duke	Early life events and impact on social status in primates, population genetics, and epigenetics	https://www.youtube.com/watch?v=q0bYUENyBAQ https://www.youtube.com/watchv=xVO03U7M0pY&index=45&list=UUntn0SbKA6sXUIMHgGXokUg
**Dr. Geerat Vermeij**	Distinguished Professor of Earth and Planetary Sciences at University of California - Davis	The evolution of mollusks, particularly coevolution between predators and prey and the impact on morphology	https://www.shapeoflife.org/video/geerat-vermeij-evolutionary-biologist-reading-shell%E2%80%99s-story https://geology.ucdavis.edu/people/faculty/vermeij
**Dr. Jessica Ware**	Associate Professor at Rutgers University, Research associate at American Museum of Natural History and the National Museum of Natural History	Dragonfly flying and mating and cockroach evolution.	https://www.futurity.org/pantala-flavescens-dragonfly-1121142-2/ https://www.youtube.com/watch?v=r-_PSJ2LeNI https://500womenscientists.org/updates/2019/3/24/so-much-left-to-learn
**Dr. Luis Zaman**	Assistant Professor in Ecology and Evolutionary Biology at University of Michigan	How evolution creates diversity and complexity and how that complexity creates a feedback loop between what evolution produces and what then becomes selectively favorable	https://www.youtube.com/watch?v=u8YN4BOUTkg
